# Parental Awareness of Oral Health and Nutritional Behavior in Children with Congenital Heart Diseases Compared to Healthy Children

**DOI:** 10.3390/ijerph17197057

**Published:** 2020-09-27

**Authors:** Nelly Schulz-Weidner, Thushiha Logeswaran, Maximiliane Amelie Schlenz, Norbert Krämer, Julia Camilla Bulski

**Affiliations:** 1 Department of Pediatric Dentistry, Dental Clinic, Justus Liebig University, Schlangenzahl 14, 35392 Giessen, Germany; norbert.kraemer@dentist.med.uni-giessen.de (N.K.); julia.c.bulski@dentist.med.uni-giessen.de (J.C.B.); 2 Centre for Heart Surgery, Medical Centre of Pediatrics, Justus Liebig University, Feulgenstrasse 12, 35394 Giessen, Germany; thushiha.logeswaran@paediat.med.uni-giessen.de; 3 Department of Prosthodontics, Dental Clinic, Justus Liebig University, Schlangenzahl 14, 35392 Giessen, Germany; maximiliane.a.schlenz@dentist.med.uni-giessen.de

**Keywords:** oral prevention, oral health, congenital heart disease, dental care, infective endocarditis, questionnaire, parents

## Abstract

Parents of children with congenital heart disease (CHD) seem to underestimate the importance of optimized oral health. The low priority for a good oral hygiene and a healthy diet can be a risk factor for odontogenic bacteremia and infective endocarditis. The aim of this study was the evaluation of the disease awareness and dental knowledge of the parents using a questionnaire. Therefore, parents from 107 children with CHD and a healthy control group (HCG) consisting of 101 children both aged 2 to 6 years were asked to complete a questionnaire containing items about the general health, oral hygiene behavior, preventive measures, dental visits and intake of potential drinks and cariogenic nutrition of their child. The results of the present study show that the CHD group had a poorer oral health behavior than the HCG. Healthy children brushed their teeth significantly more often (65.4%) than the CHD children (45.1%). Only 75% of CHD children used fluorides in their daily life in comparison to 86.6% of the healthy children, 8.7% of their parents neglected completely fluoride supplementation. Of all CHD children 23.1% in comparison to 8.1% of the controls had never visited a dentist before. Furthermore, the daily consumption of cariogenic food and drinks was generally higher in the CHD group. These findings demonstrate a need for improvement in parental knowledge of the efficiency of different measures to improve dental health. This important oral health for CHD children from the early stage of life is obvious, especially regarding their risk for odontogenic bacteria and infective endocarditis.

## 1. Introduction

Children with congenital heart defects (CHD) are predisposed to develop oral diseases [1,2,3]. Studies have shown increased poor oral hygiene and caries risk in children with CHD. In addition, the prevalence of caries in children with CHD is significantly higher than in healthy children [4,5]. Sivertsen et al. found that this impairment of oral health status has systemic dangerous effects in one third of these children, especially episodes of increased bacteremia [3,6]. With regard to possible dental sepsis and the risk of endocarditis, untreated carious teeth should be avoided in children with congenital heart defects [7]. However, experience has shown that young patients do not present themselves until a very advanced stage of carious disease, so that dental restoration can often only be performed under general anesthesia (GA). This is also shown by other studies, in which 83% of dental restorations were performed under GA [8,9]. Rehabilitation under GA poses an additional problem for these high-risk children, since anesthesia is associated with an increased risk even in the presence of a general disease [2,10].

Early intervention can be a useful tool to optimize the oral health status of this specific group of children, including the prevention of infective endocarditis. Unfortunately, many parents are not aware of this particular problem [11]. Although parents seem to have an excellent knowledge of their children’s existing heart disease and the necessary medications, they underestimate the potential risk of endocarditis [12]. Contrary to these findings, Balmer et al. reported excellent knowledge of their parents’ oral health and endocarditis, while their children’s dental health was still poor [11].

One of the main reasons for the neglect of oral health appears to be the child’s heart disease, which plays a central role in the everyday life of the affected children, making it difficult to implement age-appropriate oral hygiene and dietary factors [13,14]. In addition, hospitalization of children seems to interfere with the normal dental check-ups, which is exacerbated by the lack of experienced pediatric dentists who are able to treat these special patients with their increased medical risk [2,15].

Preliminary studies have shown that disease awareness and dental knowledge in families with children with CHD is inadequate in relation to their needs and is overall worse than in families without CHD [2,16]. In agreement with these authors, Pimentel et al. again showed that oral health awareness in families with children with CHD is insufficient and underestimated [17]. Balmer et al. reported that only 79% of children with CHD had ever consulted a dentist, only 29% received oral health instructions and only 13% received advice on fluoride supplementation. Moreover, only 64% of parents were aware of the importance of oral health in CHD [11]. All these results are consistent with other studies that describe that less than 30% of children with CHD received professional advice on preventive dental care. In addition, only 16% received advice on the use of fluoride supplementation. This lack of information from parents seems to be an important aspect to be addressed to improve oral health in the future [1,4,18].

However, to the best of our knowledge, the parents’ awareness of CHD children compared to healthy controls has not yet been investigated. Therefore, we wanted to identify possible causes and to determine whether there is indeed a difference in the oral health behavior of parents of CHD children compared to healthy children.

Using a questionnaire, we evaluated the frequency and performance of oral brushing, fluoridation measures for caries prophylaxis, dental care through dental appointments, and consumption of cariogenic potential beverages and foods in CHD children compared to a healthy control group (HCG).

## 2. Materials and Methods

The prospective study was realized in accordance with the guidelines of the Declaration of Helsinki and approved by the local ethics committee of the Department of Medicine, Justus Liebig University Giessen (AZ 186/17). All parents including legal guardians of the participating children had given their written informed consent. The current study took place during the period between February 2018 and August 2019.

### 2.1. Study Participants

Pre-school children (CHD children/HCG) aged between 2 to 6 years were included in the study. Patients with various severities and types of congenital heart defects (CHD) according to the categorization of Warnes et al. [19] were allowed to participate, but only after undergoing minimum one heart operation in the past. The CHD group consisted of 107 children, who were in need for life-long follow-up at the Medical Centre for Pediatrics, Department of Pediatric Heart Surgery, Giessen University Hospital (UKGM), Germany, living with their parents at home. Among these CHD children 16 had undergone a heart transplantation (HT). Furthermore, 16 children with co-morbidities and syndromes were included in the study (e.g., trisomy 21, 22q11, Williams-Beuren-, Barth-, Turner-, Charge-syndrome). The group of patients with a high risk of serious health damage in the case of endocarditis, as recommended [20], were all in possession of an endocarditis pass. Two patients had a cleft lip and palate.

As controls, a total number of 101 children without general diseases (maximum ASA class I [21]) were included in the current study. These healthy children represented a cohort from local preschools in and nearby Giessen, Germany.

### 2.2. Data Collection and Analysis

Firstly, the dmf-t value according to the WHO criteria regarding caries history was investigated for every patient [22]. After that, all parents were asked to complete a questionnaire containing items regarding the general and special medical history, the oral health behavior as well as the food consumption of their child. The first part of the questionnaire concerning the anamnesis of the child included data about general health as well as items about the daily oral hygiene and dental prevention such as tooth brushing, recommended fluoridation measures for caries prophylaxis, dental visits and duration of breastfeeding (Questions about the medical history, daily oral hygiene and dental visits–Supplementary Part S1).

For the analysis of possible cariogenic nutrition, a second part of a well-established Food Frequency Questionnaire (FFQ–Supplementary Part S2), was used. The questions were partly taken from the validated questionnaire from the general health study of children and adolescents (KiGGS) carried out by the Robert-Koch institute, Berlin, Germany [23,24,25]. Thus, the daily consumed amount of cariogenic food and drinks could be identified. Each question of the FFQ was constructed equally with multiple choice response options, which was divided into two parts. At first, the parents had to declare the frequency of the intake (never, 1/month, 2–3/month, 1–2/week, 3–4/week, 5–6/week, 1/day, 2–3/day, 4–5/day, more often than 5/day). The second part of each question asked for the quantity of intake. Beverages were measured in 200 mL glasses (¼ glass or less, ½ glass, 1 glass, 2 glasses, 3 glasses or more). Depending on the different products the response options for the quantity of food intake were the number of pieces (e.g., fruit, cake, chocolate bar in ¼ or less, ½, 1, 2, 3 or more pieces) or tea and table spoons (½ or more, 1, 2, 3, 4 or more tea/table spoons).

For statistical analysis, data was analyzed using SPSS^®^ for Windows (version 25.0, IBM Corporation, Armonk, New York, NY, USA). Statistical differences between the information of the CHD and controls were determined using the Chi-squared-test and T-test for normally distributed values and the Mann-Whitney-U-test for not normally distributed values. The level of significance was *p* < 0.05.

## 3. Results

In total 107 children with CHD and 101 healthy children (HCG) participated in the study. The CHD group consisted of 62 boys (57.94%) and 45 girls (42.06%), meanwhile 56 boys (55.45%) and 45 girls (44.55%) participated in the control group. The mean age of the two groups was 4.63 ± 1.46 years (CHD) and 4.40 ± 1.21 years (HCG). The age between the groups was not significantly different (*p* > 0.05). The dropout rate of the CHD group was 15.2% vs. 50.5% in HCG.

**Figure 1:**
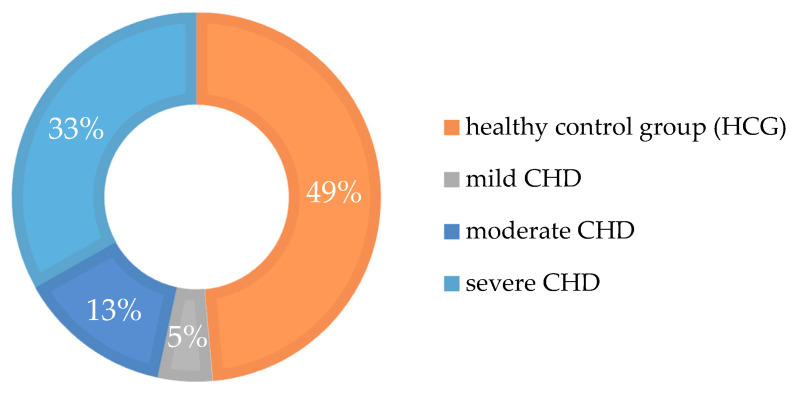
Incidence of congenital heart defects (CHD) and healthy children (HCG) in the current study. Categorization into three severities according to Warnes et al. [19].

In the CHD group, the first-mentioned diagnosis of the medical record was used to determine the severity of each children’s heart disease. Due to the lack of full information, 104 questionnaires from the CHD group and 82 from the controls could be taken into full analysis. If a combination of several heart defects and different degree of severities occurred, the most serious one was taken. Figure 1 shows all participating children, depending on the different severities of congenital cardiovascular defects (mild, moderate, severe [19]).

Determining the caries risk children of the CHD group were presented in each age group whereas only in the age group under 4 years 6.1% has an increased risk (Table 1).

Regarding the oral health care of both groups (Table 2), the frequency of daily tooth brushing varied significantly (*p* < 0.001). The HCG brushed most commonly twice a day their teeth (65.4%) compared to 45.1% of the CHD group. Of the HCG children 38 (46.3%) and 23 (22.1%) of CHD brushed even more than twice a day. Only two parents of CHD children declared, that they never or not daily brush their children’s teeth. The most common assistance during tooth brushing in both groups was the brushing of parent and child together (72.1% CHD vs. 73.2% HCG). In both groups, about 20% of the parents cleaned their children’s teeth alone (21.2% CHD vs. 18.3% HCG). The minority of the children brushed their teeth without assistance (6.7% CHD vs. 2.4% HCG). There was no significant difference concerning the supervision during the daily oral hygiene between the two groups (*p* > 0.05).

The recommended fluoridation measures for caries prophylaxis were used in 75% of the CHD and 86.6% of the control cases. Only 9 (CHD) versus 4 children (HCG) did not use fluoride (Table 3). The most common way of usage was the local fluoridation with toothpaste (69.2% CHD vs. 79.3% HCG). In 34.6% of households of CHD and 62.2% of control children utilized fluoride containing table salt. In both groups, fluoride tablets were given especially during the first year of life (22.1% CHD vs. 31.7% HCG). Furthermore, children with CHD continued with this medication until this second year of life or even longer (26.9%). With regard to a possible overdose of fluoride, no multiple applications in the systemic form (namely, fluoridated table salt and tablets) could be detected. Statistical differences of fluoride supplementation between CHD and control group could not been observed (*p* > 0.05).

Concerning the frequency of dental visits (Table 4) there was no significant difference between both groups (38.5% CHD vs. 50% HCG; Mann-Whitney-U-test, *p* > 0.05). A number of 24 CHD children (23.1%) in contrast to 10 controls (8.2%) had never been to the dentist before. Most of the legal guardians did not declare a specific reason why they have not been to the dentist with their child (70.8% CHD vs. 80% HCG). A minor part of the CHD group had also more than two dental visits per year (14.4%). 50% of the control group were supervised from a dentist in their preschool which was significant more in comparison to CHD children (Chi-squared-test, *p* < 0.01). The majority of CHD group with 58 children did not have any dental supervision in their preschool.

**Figure 2:**
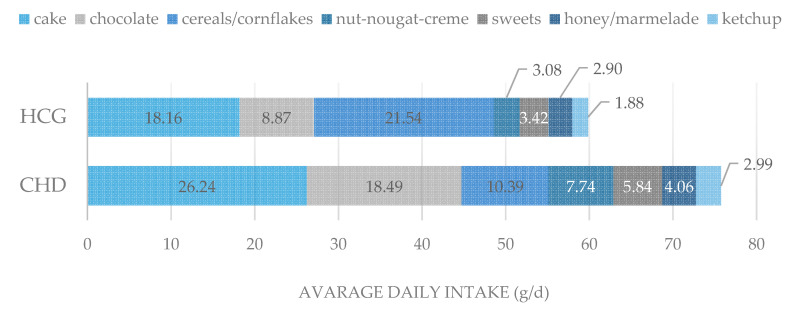
Average daily consumption of cariogenic food among CHD children and controls in gram per day (g/d).

The daily intake of caries potential food was higher in the group of CHDs with 75.76 g/d, while the controls consumed 59.85 g/d (Figure 2). CHD children ate significantly more cariogenic food, as cereals including cornflakes (21.54 g/d vs. 10.39 g/d), as well as chocolate and nut nougat cream compared to the controls (Mann-Whitney-U-test, *p* < 0.001; *p* < 0.01).

**Figure 3:**
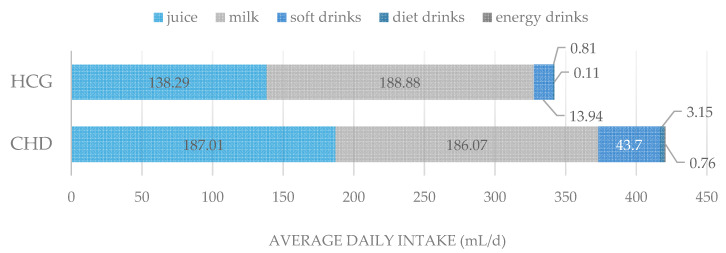
Average daily consumption of cariogenic drinks among CHD children and controls in mL per day (mL/d).

On average, the daily consumption of cariogenic drinks in the CHD group in comparison to the controls was higher (Figure 3), without significant difference (Mann-Whitney-U-test, *p* > 0.05). In both groups, the intake of milk was almost identical. The daily main drink was fruit- and vegetable juices in the CHD group and milk among the controls, which did not significantly differ.

## 4. Discussion

This study is based on a questionnaire for parents of CHD children in comparison to healthy controls. The response rate to this questionnaire within the study group can be classified as satisfactory with a percentage of 81.2% in comparison to 50.5% in the control group. We explain this difference with a lack of interest of parents with healthy children combined with the increased time required to complete the questionnaire.

As far as the frequency of consumption is concerned, the information provided by the parents must be considered carefully. As this is only an estimate, it is to be expected that the given information on quantities consumed could be either over- or underestimated by the parents.

The World Health Organization (WHO) recommends reducing the intake of free sugar to less than 10 percent energy at all stages of life to reduce the risk of tooth decay. This corresponds to no more than 50 g of sugar per day (approximately 10 teaspoons) for an average adult [27]. The daily consumption of cariogenic food was higher in the group of children with heart disease, averaging 75.76 g per day, than in healthy children (59.85 g/d). The daily consumption of cariogenic beverages in children with heart disease was on average higher than in the healthy control group. Regarding to this pointed aspect referring the caries risk, the consumption of cariogenic food was too high in both groups.

With regard to the increased caries risk, we were able to prove that in the healthy comparison there were fewer children with an increased caries risk according to DAJ criteria (German association for youth dental care) [26]. This is consistent with other studies that have also confirmed an increased incidence of caries in CHD children compared to healthy children [3,4].

Regarding oral hygiene, the daily cleaning of teeth was found to be done more frequently in the group of healthy children. In total, 65% of children with heart disease have brushed their teeth only once a day, in contrast to 44% of healthy children. The majority in both groups implemented daily oral hygiene at least twice a day (65.4% CHD vs. 45.1% HCG). Even 46.3% of the healthy children even cleaned more than twice a day their teeth. Brushing their teeth together with the parents was the most common procedure in both groups (72.1% CHD vs. 73.2% HCG). These results are consistent with other international studies [1,2,8,18,28,29,30]. In terms of oral hygiene, about 8.7% of all children with CHD brush their teeth only once a day. Nevertheless only a few CHD children did not brush their teeth daily or never [8,18,28,29]. Our figure is lower than published by Talebi et al. reporting 38% of children not brushing their teeth [31].

These differences may be due to variable approaches to oral hygiene. Besides the focus of parents and children on the general disease, long hospital stays may be the reason for the neglect of regular dental appointments and the following hygiene instructions. Another aspect could be that the parents want the children to avoid confrontations in the area of oral hygiene because of their illness.

According to the parents, the recommended fluoridation measures for caries prophylaxis in the daily routine of healthy children is far more common than in children with CHD. Due to the local guidelines of the German Society of Pediatric Dentistry at the time of data collection, we expect that the majority of the children used toothpaste with a content of 500 ppm fluoride. Since the caries protective effect of fluorides is undisputed [32], it is surprising that only 75% of children with heart disease make use of fluoride. A small number of 8.7% of parents provided their children no recommended fluoridation measures for caries prophylaxis, 16.3% could not state a reason for that. These results agree with Koerdt et al. who even found that 26.7% do not supplement fluoride in their children [18]. In each case, the primary intake of fluorides was similar (by toothpaste). The fluoride intake was supplementing of table salt containing fluoride in by 34.6% of the children with heart disease vs. 62.2% of the controls. The intake of fluoride tablets was recorded more frequently in the CHD group, over a longer period of time, up to the second half of the year and beyond. These results agree with other studies demonstrating the most frequent intake of fluoride via toothpaste [8,30], but the frequency is below in comparison to healthy volunteers [1].

In addition, the survey of parents showed that only a few children with heart disease appear regularly for a dental check-up. Of these children, 25% of these children have never been to the dentist before. Most of the parents (70.8%) stated no reason and 12.5% no time or no need for this. These data underline that regular visits to the family dentist are less frequent in children with CHD which is in fully accordance with all other published studies [1,10,11,18,28,29,30,33]. To our knowledge, this is the first study demonstrating the differences of oral hygiene routine comparing CHD children with same aged healthy controls.

Unfortunately, children with CHD are often only dental treated for existing symptoms [19,20], which means that more complex treatments often have to be carried out in advanced lesions. This means that, among other factors, the dental restorations must be performed under general anesthesia due to the advanced extensive dental diseases. This includes a risk especially for this special group of children who are vulnerable to infective endocarditis. Furthermore, a rehabilitation under general anesthesia is an additional problem for these high-risk children, since anesthesia has an increased risk anyway with existing general disease [2,10].

## 5. Conclusions

Our findings provide evidence for an unacceptable lack of knowledge about the importance of an optimized oral health among parents with CHD children compared to healthy controls. An improvement in the nutritional behavior as well as the education in prevention of dental caries, e.g., brushing routine, fluoride supplementation, regular dental check-ups is needed. Therefore, the implementation of a strict schedule for the children always in cooperation with the parents has to be established. Moreover, an interdisciplinary cooperation between pediatrics, pediatric dentists and cardiologists must be promoted to reach a better understanding of the important association of dental health and cardiac disease and the prevention of dental bacteremia and infective endocarditis.

## Figures and Tables

**Table 1:** Distribution of high risk dmf-t values in the groups of CHD compared to HCG according to the criteria of the German association for youth dental care (DAJ) [26].

Age Group	Limiting Value	CHD		HCG	
		Number of Children in Age Group	Children with High Caries Risk	Number of Children in Age Group	Children with High Caries Risk
<3 years	dmf-t > 0	18	1 (5.6%)	13	0 (0%)
<4 years	dmf-t > 2	23	2 (8.3%)	33	2 (6.1%)
<5 years	dmf-t > 4	20	2 (10%)	18	0 (0%)
<6–7 years	dmf-t > 5	46	6 (13%)	37	0 (0%)
Total		107	11 (10.3%)	101	2 (2%)

**Table 2:** Answers concerning the daily oral hygiene of the child with congenital heart disease (CHD) compared with the healthy control group (HCG).

Daily Oral Hygiene	CHD		HCG	
	*n* = 104	%	*n* = 82	%
Frequency of Tooth Brushing				
- never	2	1.9	0	0
- 1/day	9	8.7	2	2.4
- 2/day	68	65.4	37	45.1
- >2/day	23	22.1	38	46.3
- no answer	2	1.9	5	6.1
Supervision				
- child alone	7	6.7	2	2.4
- parents alone	22	21.2	15	18.3
- both together	75	72.1	60	73.2
- no answer	0	0	5	6.1

**Table 3:** Recommended fluoridation measures for caries prophylaxis within the CHD and healthy control group (HCG).

Fluoridation Measures for Caries Prophylaxis	CHD		HCG	
	*n* = 104	%	*n* = 82	%
Yes	78	75.0	71	86.6
fluoridated toothpaste	72	69.2	65	79.3
- no answer	24	23.1	5	6.1
fluoridated salt	36	34.6	51	62.2
- no answer	30	28.9	7	8.5
fluoride tablets	64	61.5	34	41.5
- during 1st year of life	23	22.1	26	31.7
- ≤2nd year of life or longer	20	19.2	7	8.5
- to this day	8	7.7	1	1.2
- no answer	31	29.8	11	13.4
No	9	8.7	4	4.9
No answer	17	16.3	7	8.5

**Table 4:** Answers regarding the regularity of dental visits in the CHD group compared with HCG.

Regularity of Dental Visits	CHD		HCG	
	*n* = 104	%	*n* = 82	%
Dental visits				
- never	24	23.1	10	12.2
- less than once a year	11	10.6	0	0
- 1/year	14	13.5	23	28.1
- 2/year	40	38.5	41	50.0
- more than 2/year	15	14.4	1	1.2
- no answer	0	0	7	8.5
Reasons why children have never been to the dentist before				
- lack of time	3	12.5	0	0
- no need	3	12.5	1	10.0
- child’s anxiety	1	4.2	1	10.0
- another reason/no answer	17	70.8	8	80.0
Support by a dentist in pre-school				
- Yes	38	36.2	41	50.0
- No	58	55.2	26	31.7
- No answer	9	8.6	15	18.3

## Supplementary Materials

The following are available online at https://www.mdpi.com/1660-4601/17/19/7057/s1, Part S1. Questions about the medical history and daily oral hygiene and dental visits; Part S2. Food Frequency Questionnaire (FFQ). Questions are partly taken from the validated questionnaire from the general health study of children and adolescents (KiGGS). Credits to the Robert Koch-Institute, Berlin, Germany.
