# Supplementary material for: Parental Awareness of Oral Health and Nutritional Behavior in Children with Congenital Heart Diseases Compared to Healthy Children
Source: Int J Environ Res Public Health. 2020 Sep 27;17(19):7057. doi: 10.3390/ijerph17197057 (PMC7579597; doi:10.3390/ijerph17197057)
Supplement: Supplementary file 1 [file ijerph-17-07057-s001.pdf]

International Journal of
Environmental Research and Public Health

MDPI

Part S1. Questions about the medical history and daily oral hygiene and dental visits

1. Does your child take medication on a daily basis?
   - Yes.
   - No.
   - No answer.
2. Do you have a document for endocarditis prophylaxis for your child?
   - Yes.
   - No.
   - No answer.
3. How long was your child breastfed?
   - Not at all.
   - ≤6 months
   - ≤12 months
   - >12 months
   - No answer.
4. How often does your child brush his/her teeth?
   - Never/not daily.
   - 1/day
   - 2/day
   - >2/day
   - No answer.
5. How does your child brush his/her teeth?
   - Alone.
   - Only parents.
   - Child and parent together.
   - No answer.
6. Does our child receive fluoride supplementation?
   - No.
   - Yes.
     - fluoridated toothpaste
     - fluoridated table salt
     - fluoride tablets
       - until 1st year of life
       - until 2nd year of life or longer11
       - to this day
       - No answer.
   - No answer.
7. How often does your child visit a dentist?
   - Never.
   - Less than 1/year.
   - 1/year
   - 2/year
   - More often.
   - No answer.
8. If no, why not?
   - Lack of time.
   - No need.
   - Child's anxiety.
   - Another reason / no answer.
9. Does a dentist supervise your child's kindergarten?
   - Yes.
   - No.
   - No answer.

Part S2. Food Frequency Questionnaire (FFQ). Questions are partly taken from the validated questionnaire from the general health study of children and adolescents (KiGGS). Credits to the Robert Koch-Institute, Berlin, Germany.

1a. How often did your child drink milk (including milk for cereals, cocoa)?
- never
- 1/month
- 2–3/month
- 1–2/week
- 3–4/week
- 5–6/week
- 1/day
- 2–3/day
- 4–5/day
- >5/day

1b. When your child drinks milk, how much does he/she drink?
- ≤¼ glass
- ½ glass
- 1 glass (200 mL)
- 2 glasses
- ≥3 glasses

2a. How often did your child drink soft drinks (e.g. coke, lemonade, iced tea)?
- never
- 1/month
- 2–3/month
- 1–2/week
- 3–4/week
- 5–6/week
- 1/day
- 2–3/day
- 4–5/day
- >5/day

2b. When your child drinks soft drinks, how much does he/she drink?
- ≤¼ glass
- ½ glass
- 1 glass (200 mL)
- 2 glasses
- ≥3 glasses

2c. How often were the soft drinks diet products?
- never
- ¼ of the consumption
- ½ of the consumption
- ¾ of the consumption
- always

3a. How often does your child drink energy drinks?
- never
- 1/month
- 2–3/month
- 1–2/week

- 3–4/week
- 5–6/week
- 1/day
- 2–3/day
- 4–5/day
- > 5/day

3b. When your child drinks energy drinks, how much does he/she drink?
- ≤¼ glass
- ½ glass
- 1 glass (200 mL)
- 2 glasses
- ≥3 glasses

4a. How often did your child drink fruit or vegetable juices?
- never
- 1/month
- 2–3/month
- 1–2/week
- 3–4/week
- 5–6/week
- 1/day
- 2–3/day
- 4–5/day
- >5/day

4b. When your child drinks juices, how much does he/she drink?
- ≤¼ glass
- ½ glass
- 1 glass (200 mL)
- 2 glasses
- ≥3 glasses
- ≤¼ glass
- ½ glass
- 1 glass (200 mL)
- 2 glasses
- ≥3 glasses

5a. How often did your child eat fresh fruit?
- never
- 1/month
- 2–3/month
- 1–2/week
- 3–4/week
- 5–6/week
- 1/day
- 2–3/day
- 4–5/day
- >5/day

5b. When your child ate fresh fruit, how much did he/she eat?
- ≤¼ portion or ¼ bowl
- ½ portion or ½ bowl
- 1 portion or 1 bowl
- 2 portions or 2 bowls
- ≥3 portions or 3 bowls

6a. How often did your child eat honey or marmalade?
- never
- 1/month
- 2–3/month
- 1–2/week
- 3–4/week
- 5–6/week
- 1/day
- 2–3/day
- 4–5/day
- >5/day

6b. When your child ate honey or marmalade, how much did he/she eat?
- ≤½ teaspoon
- 1 teaspoon
- 2 teaspoons
- 3 teaspoons
- 4 teaspoons

7a. How often did your child eat nut nougat creme?
- never
- 1/month
- 2–3/month
- 1–2/week
- 3–4/week
- 5–6/week
- 1/day
- 2–3/day
- 4–5/day
- >5/day

7b. When your child ate nut nougat creme, how much did he/she eat?
- ≤½ teaspoon
- 1 teaspoon
- 2 teaspoons
- 3 teaspoons
- 4 teaspoons

8a. How often did your child eat cereals or cornflakes?
- never
- 1/month
- 2–3/month
- 1–2/week
- 3–4/week
- 5–6/week
- 1/day
- 2–3/day
- 4–5/day
- >5/day

8b. When your child ate cereals or cornflakes, how much did he/she eat?
- ≤¼ bowl
- ½ bowl
- 1 bowl
- 2 bowls
- ≥3 bowls

9a. How often did your child eat ketchup or mayonnaise?
- never
- 1/month
- 2–3/month
- 1–2/week
- 3–4/week
- 5–6/week
- 1/day
- 2–3/day
- 4–5/day
- >5/day

9b. When your child ate ketchup or mayonnaise, how much did he/she eat?
- ≤½ tablespoon
- 1 tablespoon
- 2 tablespoons

- 3 tablespoons
- ≥4 tablespoons

10a. How often did your child eat cake or pastries?
- never
- 1/month
- 2–3/month
- 1–2/week
- 3–4/week
- 5–6/week
- 1/day
- 2–3/day
- 4–5/day
- >5/day

10b. When your child ate cake or pastries, how much did he/she eat?
- ≤¼ piece of cake
- ½ piece of cake
- 1 piece of cake
- 2 pieces of cake
- ≥3 pieces of cake

11a. How often did your child eat chocolate or chocolate bars?
- never
- 1/month
- 2–3/month
- 1–2/week
- 3–4/week
- 5–6/week
- 1/day
- 2–3/day
- 4–5/day
- >5/day

11b. When your child ate chocolate or candy bars, how much did he/she eat?
- ≤ ½ small candy bar
- ¼ bar of chocolate or 1 small candy bar
- ½ bar of chocolate or 1 big candy bar
- 1 bar of chocolate or 2 big candy bars
- ≥2 chocolate bars

12a. How often did your child eat other sweets (chips, gummy bears etc.)?
- never
- 1/month
- 2–3/month
- 1–2/week
- 3–4/week
- 5–6/week
- 1/day
- 2–3/day
- 4–5/day
- >5/day

12b. When your child ate sweets, how much did he/she eat?
- 1 piece
- 2–5 pieces
- 6–10 pieces
- 11–20 pieces
- ≥21 pieces
